# New insights into the transport processes controlling the sulfate-methane-transition-zone near methane vents

**DOI:** 10.1038/srep26701

**Published:** 2016-05-27

**Authors:** Nabil Sultan, Sébastien Garziglia, Livio Ruffine

**Affiliations:** 1 Ifremer, REM/GM/LAD, Institut Carnot Ifremer-EDROME, France

## Abstract

Over the past years, several studies have raised concerns about the possible interactions between methane hydrate decomposition and external change. To carry out such an investigation, it is essential to characterize the baseline dynamics of gas hydrate systems related to natural geological and sedimentary processes. This is usually treated through the analysis of sulfate-reduction coupled to anaerobic oxidation of methane (AOM). Here, we model sulfate reduction coupled with AOM as a two-dimensional (2D) problem including, advective and diffusive transport. This is applied to a case study from a deep-water site off Nigeria’s coast where lateral methane advection through turbidite layers was suspected. We show by analyzing the acquired data in combination with computational modeling that a two-dimensional approach is able to accurately describe the recent past dynamics of such a complex natural system. Our results show that the sulfate-methane-transition-zone (SMTZ) is not a vertical barrier for dissolved sulfate and methane. We also show that such a modeling is able to assess short timescale variations in the order of decades to centuries.

The sulfate-methane transition zone (SMTZ) corresponds to the sedimentary interval characterized by a mutual depletion of methane and sulfate due to the microbial anaerobic oxidation (AOM) of methane^1^,^2^. The degradation of organic matter is another process responsible for the consumption of sulfate in marine sediments. However, at methane-rich cold seeps, it is usually considered as negligible compared to AOM^3^,^4^. Some authors consider that AOM plays an important role in global climate change since it buffers the transfer of methane from deep sources to the seafloor, and moderates gas flux from the ocean to the atmosphere^5^,^6^,^7^,^8^. The SMTZ is also the interval where carbonate precipitates due to the release of bicarbonates from AOM^9^,^10^,^11^. In the recent years and for several cold seep sites around the world, carbonates and pore-water sulfate profiles were used as a proxy for methane migration, including methane sourced from the decomposition of methane hydrates. For example, it has been shown that dissolved sulfate depletion can be used to quantify methane fluxes and/or gas hydrate saturation^12^. Other studies have developed back-calculation from pore-water sulfate profiles to associate sedimentary processes and their timing to transient geochemical conditions^13^, or to develop quantitative analyses of mass transport deposits^14^. All the previously cited references and supporting evidence are based on a vertical analysis of sulfate profiles. While it is recognized that vertical analysis can provide valuable insight into fluid transport and reactions through layered sediments, more advanced investigation including horizontal processes (through source terms or 2-D calculations) is needed to accurately reflect complex conditions where vertical processes are altered by the presence of lithological discontinuities and heterogeneously distributed gas hydrates.

## Results

### Data: complex free gas/gas hydrate system

**Figure 1:**
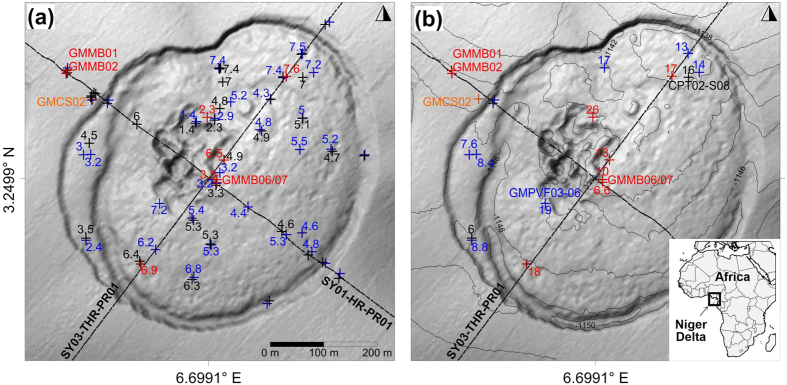
Top and base of GHOZ. Overview of stations and acquired data. Calypso core: orange crosses; MeBo drills: red crosses; Penfeld piezocone: black crosses; Penfeld celerimeter: blue crosses; projected on shaded bathymetry maps of the studied pockmark. Only sites and data used in the present paper are labeled. Locations of SYSIF seismic profiles SY01-HR-PR01 and SY03-THR-PR01, are also shown. The top (in panel (**a**)) and base (in panel (**b**)) of GHOZ whenever identified by coring or *in-situ* measurements are indicated. Maps created using Surfer 11.6 (http://www.goldensoftware.com/surfer-version-info).

**Figure 2:**
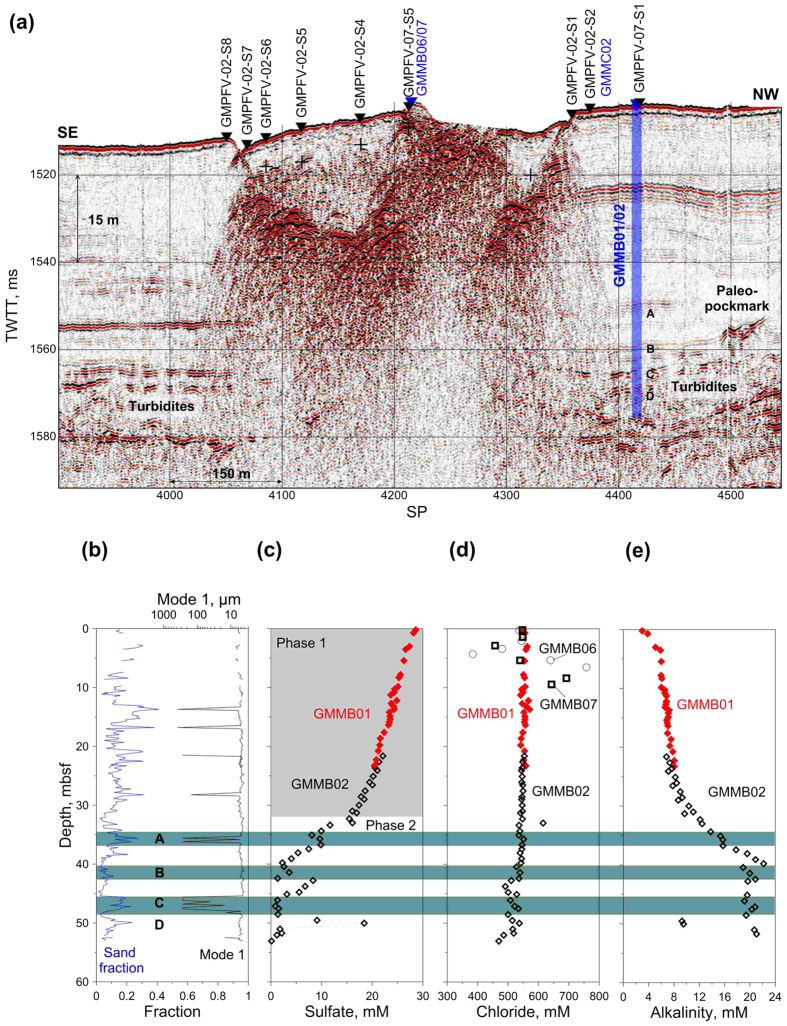
Geophysical, geochemical and sedimentological data. (**a**) Seismic profiles SY01-HR-PR01 showing a significant contrast between high-amplitude chaotic facies at the center of the pockmark and low-amplitude subparallel reflectors of surrounding sediments. Four MeBo drill sites, 1 Calypso core and 9 *in-situ* Penfeld celerimeter measurements were used to define the top of the gas hydrates (black dash line and black crosses) as indicated on the seismic line. Level A corresponds to the presence of carbonate foraminifera while levels B, C and D correspond to high amplitude turbidite layers. A paleo-pockmark overlaying these turbidite layers is also indicated in (**a**). (**b**) Sand fraction and grain size distribution mode 1^22^ as a function of depth obtained from drill sites GMMB01 and GMMB02 showing that levels A, B, C and D correspond to high sand (or sand-like for layer A) fractions. (**c**) Concentration of sulfate in pore water from GMMB01 (red diamonds) and GMMB02 (black diamonds). The sulfate profile in (**c**) appears to result from two different methane advection-diffusion phases (phase 1 and phase 2). (**d**) Concentration of chloride in pore water from GMMB01, GMMB02, GMMB06 and GMMB07 and (**e**) alkalinity in pore water from GMMB01 and GMMB02.

The present study focuses on an area located at around 1120 m water depth offshore Nigeria and where 2D advection and diffusion processes are suspected^15^ to operate. The sea-floor morphology is marked by the presence of a 600 m diameter ring-like depression referred to as pockmark A^16^ (Fig. 1). This morphological feature delineates a shallow methane hydrate accumulation that can be classified as a high gas flux (HGF) system^17^,^18^ based on seismic evidence of faulting, which provides pathways for deep migrating allochtonous gas^15^. Additional evidence for high gas flux and complex dynamical interactions were taken from the coexistence of free gas within the gas hydrate occurrence zone (GHOS) and, most particularly, from the presence of methane hydrates with a bubble-type fabric^15^. Hydrates with such a fabric were recovered in between 1 and 2 m depth beneath a site of active seafloor venting, near the center of pockmark A^15^. Figure 1a reveals that in this sector hydrate accumulation is the shallowest and the thickest according to *in situ* acoustic and strength measurements, as well as to the analyses of MeBo^19^ drill cores. Outside this sector, the top of the gas hydrate occurrence zone (GHOZ) is located at depths ranging from 2.3 to 7.6 m below seabed, with a tendency to deepen towards the periphery of pockmark A^20^ (cf. Supplementary information). On near-seabed seismic data, a change from an upper zone of low reflectivity to an underlying zone characterized by moderate to high-amplitude, chaotic reflection patterns is consistent with the top of hydrate occurrence inferred from *in situ* measurements and core analyses (Fig. 2). More commonly, the brightest reflections have a reversed polarity and correspond to apexes of diffraction hyperbolae (Fig. 2). By analogy with the arguments put forward by Wood and co-authors^21^, these seismic features are thought to be caused by free gas accumulations along fractures. The majority of *in situ* geotechnical measurements reported in Fig. 1 halted on gas hydrates preventing penetration down to the maximum depth of 30 mbsf. Based on the few *in situ* measurements that reached this depth and on drill core analyses, the base of the gas hydrate and free gas occurrence zone is located at depths varying from 6 to 26 m below seabed (Fig. 1). This irregular base is less precisely defined on the seismic data than the top of the GHOZ, probably because of transmission losses and scattering through a complex subsurface network of hydrate filled fractures partially trapping free gas (Fig. 2). It is however noteworthy that Wei and co-authors^20^ have not reported any evidence of gas hydrates or free gas below the diffuse base of the zone characterized by moderate to high-amplitude, chaotic reflection patterns as outlined in Fig. 2.

Sedimentological studies and grain size analyses at site GMMB01/GMMB02 reveal the presence of several coarse-grained intercalations (primary mode^22^ between 63 and 300 micrometers) in a clay sequence (Fig. 2b). Core-seismic correlation shows that the three lowermost coarse-grained intercalations correspond to the presence of turbidite layers (Fig. 2b) while the upper sub-parallel reflectors (above 40 mbsf) are regional and are an indicator of the presence of carbonate foraminifera.

Sulfate and chloride analyses carried out at several depths within GMMB01 and GMMB02 indicate an important decrease in sulfate concentrations at the level of layers A to D and a small change in chloride concentrations at the level of layers B to D (Fig. 2c,d). By contrast, the erratic chloride data measured from cores GMMB06 and GMMB07 (Fig. 2b) are indicators of hydrate dissociation upon core recovery (low chloride values) and recent *in situ* hydrate formation (high chloride values)^18^.

Potential causes of the GMMB02 S-shaped sulfate profile shown in Fig. 2c include (i) submarine landslides and mass transport deposits^14^, (ii) lateral migration of methane-poor fluid through turbidite layers or (iii) sediment pore water contamination from sea-water during drilling. However, acquired data do not support the previous three hypotheses. Indeed, no morphological or sedimentological evidence for any landslide were found to support the first hypothesis and low sulfate values within turbidite layers do not match with methane-poor fluid circulations hypothesis (low sulfate concentrations fit with coarse grain layers). Finally, the fact that at site GMMB02 low sulfate values correlate with low chloride values (Fig. 2d) and high alkalinity values (Fig. 2e) are not compatible with seawater contamination (low sulfate concentrations fit with high alkalinity values). The pore water sulfate, chloride and alkalinity data acquired from GMMB01 and GMMB02 and presented in Fig. 2c–e seem more to be the result of a complex advection/diffusion transport processe controlling the sulfate-methane-transition-zone near methane vents.

### Working hypothesis and modelling

In the following, the working hypothesis is that the GMMB01 and GMMB02 pore-water sulfate/chloride data are the result of two distinct advection-diffusion phases. The first phase (phase 1 in Fig. 2c) revealed by sulfate values decreasing smoothly with depth (above 32 mbsf in Fig. 2c) would result from a quasi-permanent diffusion regime of methane emanating from the base of the deep turbidite layers shown in Fig. 2a. Because marked drops in sulfate values correlate well with the presence of turbidite layers, the lower part of the sulfate curve (below 32 mbsf in Fig. 2c) is considered to result from lateral advection of methane-rich fluid through different permeable layers.

In order to test this hypothesis, we performed a 2D numerical modeling of sulfate reduction in methane-rich sediments. We focused on the AOM reaction as it is the main process responsible for sulfate reduction for this pockmark. Indeed, sulfate consumption due to organic matter degradation was neglected because of the seawater-like concentration of dissolved sulfate measured at the uppermost part of the core. Since free gas is confined in the central part of the pockmark and isolated from the surrounding sediment by gas hydrates, and the AOM is restricted to the liquid phase, the developed model is limited to the mass conservation of the liquid phase. Both methane (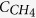) and sulfate (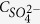) concentrations are calculated by solving a 2D advection-diffusion equation for conservative solute transport in porous media^23^. A 2D model was preferred over an asymmetric one as there is neither geological nor geochemical evidence of radial fluid advection.

We model sulfate reduction in methane-rich sediments by solving the following two differential equations:


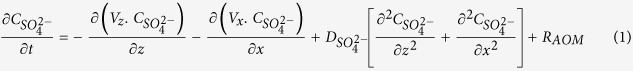



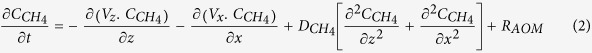


In (1) and (2), the molecular diffusion coefficient of methane (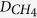) and sulfate (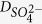) are constant (Table 1). Given the uncertainties surrounding the mechanisms controlling the diffusion/advection process, the effect of tortuosity on the molecular diffusion coefficients^24^ was considered to be of second-order (cf. Supplementary information) and was thus neglected. The advection is considered through horizontal (*v*_*x*_) and vertical (*v*_*z*_) velocity components. The AOM is taken into account in the equations (1) and (2) through the kinetic term R_AOM_ (AOM rate) given in (3). R_AOM_ depends mainly on the rate constant of anaerobic oxidation of methane, *k*_*AOM*_.


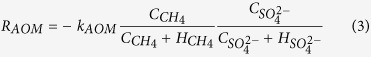


In (3), 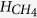 and 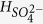 are the half-saturation constants with respect to methane and sulfate, respectively^23^. Equations (1, 2, 3) were numerically solved by approximating all the derivatives by finite differences and by using an explicit numerical method.

**Figure 3:**
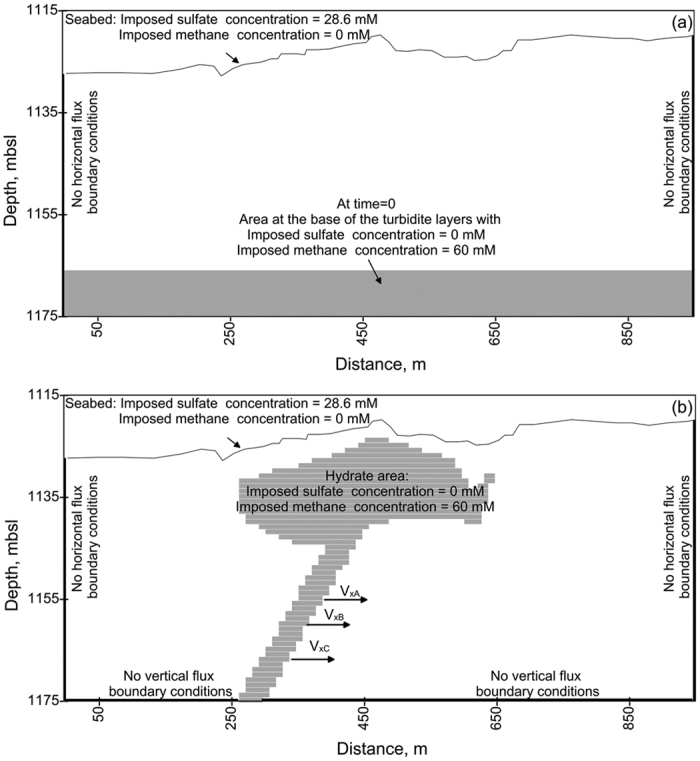
Initial and boundary conditions. Summary of initial- and boundary-conditions considered in the phase 1 (**a**) and phase 2 (**b**) calculations.

For the phase 1 calculation, the initial conditions correspond to a methane concentration of 60 mM (solubility of the methane in equilibrium with hydrates at the *in situ* temperature and pressure conditions) localized at the base of the turbidite layers shown in Fig. 2a. This initial methane concentration is allowed to decrease by diffusion during the first phase. No normal flux boundary conditions are considered at the boundary of the calculation, and sulfate and methane concentrations at the seabed were taken equal to 28.6 and 0 mM, respectively (Fig. 3a). For the phase 2 calculation, methane concentration is taken equal to 60 mM at the border of the GHOZ in the central part of the pockmark and methane-rich fluid advection is allowed to take place laterally through several turbidite layers (arrows in Fig. 3b). The horizontal fluid velocity v_x_ is considered to be variable through the permeable turbidite layers. The boundary conditions for phase 2 calculation are presented in Fig. 3b.

It is important to mention that the time gap between phase 1 and phase 2 is unknown and this corresponds to the formation of the gas-hydrate pockmark. Recently, application of uranium-thorium dating methods to authigenic carbonates recovered from this pockmark shows that seep carbonates associated with the studied pockmark activities precipitated between 13.0 and 2.5 kyr^25^. The present work did not include this intermediate phase corresponding to the pockmark formation and therefore the phase 1 calculation must be considered as an artificial technique to reproduce the upper smooth GMMB01/02 sulfate values before the phase 2 advection phase. In other words, we focused on the time elapsed since the development of the lateral migration of methane-rich fluid, and not on the age of pockmark formation.

**Figure 4:**
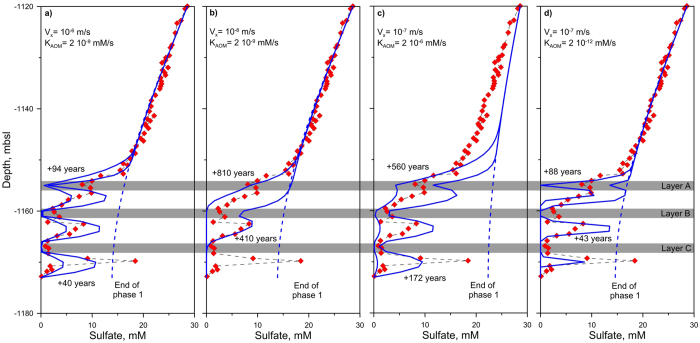
Parametric studies. Comparison of model results (blue lines) considering 4 different set of parameters with observed sulfate data (red diamonds) from sites GMMB01 and GMMB02. Blue dash-dot lines correspond to the end of phase 1. Values of v_x_ between 10^−6 ^m/s and 10^−8 ^m/s and k_AOM_ between 2 10^−6 ^and 2 10^−12 ^mM/s were considered.

A sensitivity analysis involving twenty sets of parameters was carried out in order to evaluate the influence of v_x_ through the 3 turbidite layers and k_AOM_, two main parameters affecting the results of equations (1) and (2). Figure 4 shows, for 4 different cases, the influence of those two parameters on the calculated sulfate profile at three different time steps: at the end of phase 1 and at two different time steps providing upper and lower bounds to the data obtained on GMMB01 and GMMB02. Values of v_x_ (between 10^−6 ^m/s and 10^−8 ^m/s) and k_AOM_ (between 2 10^−6^ and 2 10^−12 ^mM/s) were constrained by fitting the model results to observational data. Results from Fig. 4 show that for the highest v_x_ value (10^−6 ^m/s–Fig. 4a), sulfate concentrations fall rapidly to zero at the level of layer A while for the lowest v_x_ value (=10^−8^ m/s–Fig. 4b) indicating diffusion rather than diffusion/advection process, numerical calculations fail to reproduce localized sulfate data within turbidite layers. For high (=10^−6 ^mM/s) and low (=10^−12 ^mM/s) k_AOM_ values, the modeling results either underestimate (Fig. 4c) or overestimate (Fig. 4d) the sulfate data.

**Figure 5:**
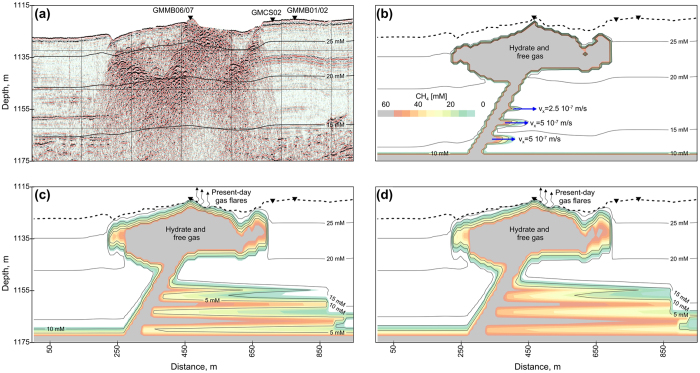
2D modeling of sulfate-reduction coupled to AOM. In panels (**b–d**), the color scale corresponds to methane concentrations, while the contour lines indicate dissolved sulfate concentrations. Panel (**a**) corresponds to the final stage of phase 1 while the three other panels correspond to (**b**) 2.5 years, (**c**) 80 years and (**d**) 130 years of methane and sulfate evolution after the initiation of lateral advection of methane (blue arrows in (**b**). Present-day gas flares indicated in panels (**c**) and (**d**) were detected by the vessel multibeam echosounder^15^.

The four graphs presented in Fig. 5 are based on a ‘trial and error’ approach to fit with the sulfate data obtained from site GMMB01 and GMMB02. The color scale in Fig. 5 corresponds to methane concentrations and contour lines indicate the dissolved sulfate concentrations. In Fig. 5, panel (a) corresponds to the final stage calculation of phase 1 where methane was completely dissolved and sulfate concentrations follow a linear trend with depth. Thirty kyr were needed to fit with the shallow dissolved sulfate profile as measured at sites GMMB01 and GMMB02. The phase 1 calculation was also used to quantify the rate constant for anaerobic methane oxidation, k_AOM_, a first-order parameter for the reaction.

For phase 2, the presence of gas-hydrate in the central part of the pockmark and the lateral advection of the methane-rich fluid are considered (Fig. 4b). A sensitivity analysis was done to investigate the effect of the v_x_ values through the four permeable layers on the model results (values in Fig. 5b). The three panels in Fig. 5 correspond to (b) 2.5 years, (c) 80 years and (d) 130 years of methane and sulfate evolution after the setting up of lateral advection of methane-rich fluid.

## Discussion

**Figure 6:**
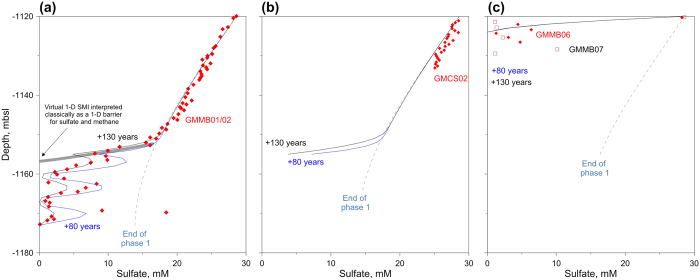
Sulfate profiles: model versus data. Comparison between model results (blue lines) and the measured sulfate concentrations (red diamonds and black squares) at 3 selected sites. Blue dash-dot lines correspond to the end of phase 1, while the blue dashed and continuous lines correspond respectively to 80 and 130 years after the lateral advection of methane.

Comparison between measurements and modeling in Fig. 6, confirms that the upper 32 mbsf of the sulfate profile at site GMMB01 and GMMB02 is shaped by the steady-state like regime (phase 1) due to the presence of methane-rich fluid within the turbidite layers. Around 30 kyr was needed to reach the measured sulfate profile considered as the end of phase 1 in Fig. 6. Between 32 mbsf and 53 mbsf, the lateral advection of methane-rich fluid through the turbidite layers (v_x_ between 2.5 10^−7 ^m/s and 5 10^−7 ^m/s) strongly influenced the sulfate profile at GMMB02. The driving factor of this relatively high lateral advection velocity seems to be related to an over-pressured intermediate gas reservoir (around 300 ms-TWTT below the seabed^15^) rather than to the hydrate dissolution/dissociation^26^ processes where the methane advection velocity is expected to be much lower than the calculated one (between 1.5 10^−9 ^m/s and 9 10^−9 ^m/s for the Cascadia margin^27^). This over-pressured intermediate gas reservoir was shown to be directly connected to a continuous gas flare reaching 500 m above the seafloor and was also suspected to feed through fractures shallow gas pockets detected thanks to the MeBo drilling^15^. Taken together, the acquired data and modeling results point out that the lateral advection process occurred only few decades ago. However, this relatively recent advection can be related to a long-term cyclic process where high and low advection velocities alternate. For period of zero advection velocity, the sulfate profile is expected to tend again to the end of phase 1 sulfate profile.

A comparison between modeled and measured sulfate data at site GMCS02 presented in Fig. 6 shows that the advection process (phase 2) has not modify yet the upper part of the sulfate profile shaped during the phase 1. Similar comparisons for sites GMMB06 and GMMB07 (Fig. 6) confirms that the hydrate occurrence in the uppermost part of the sedimentary column (less than 3 mbsf) drastically decreased the pore water sulfate concentrations. Here the model does not reproduce the sulfate oscillations measured at sites GMMB06 and GMMB07 which have been ascribed to seawater contamination. For both MeBo cores, the gas-hydrates were very close to the seabed and therefore the sulfate concentrations were expected to be below the detection limit. Indeed, gas hydrate dissolution has caused sediment expansion and expulsion outside the core-liners (recorded with the MeBo camera) and in some cases pore water exchange with sea water.

Investigation of pore water sulfate concentrations measured at 3 drilling sites in combination with computational modeling of AOM-related processes illustrates the important need to consider horizontal processes on the sulfate-methane transition zone in the investigated area. Focusing solely on vertical analysis could overshadow an important part of the process in such a complex geological systems. Indeed, the determination of the 1-D SMTZ as it is shown in Fig. 6 will lead to wrong interpretation concerning the sulfate data which are generally considered equal to zero below this virtual interface. The proposed model was able to simulate the upper quasi-linear part of the sulfate profiles in Fig. 6. Another important process controlling the pore water sulfate concentrations and leading to the 3 successive peaks in Fig. 6 was shown to be associated with lateral methane advection through permeable layers rather than a fluctuation of the sulfate and methane concentrations due to external changes^14^,^28^. The different measured sulfate profiles were of great importance to characterize the transport processes and to assess the timescale over which they took place: It was shown that lateral methane advection might have occurred some decades ago. Over longer-time scale, methane diffusion will smooth the sulfate curve (Fig. 4c). It was also shown that methane diffusion from the 3 turbidite layers cannot alone explain observational data since diffusion would generate flatter sulfate profiles (Fig. 4b).

## Methods

### Geochemical analysis using Rhizon pore water extraction

After recovery, the Calypso core was immediately cut into sections of 1m length, while the 2.52 m-length MeBo section was kept as such. The whole round sediment sections were capped and transported to the shipboard laboratory at 4 °C for pore water extraction. The latter was performed with Rhizon^29^ soil-moisture samplers, a hydrophilic, porous polymer capillary of 2.5 mm in diameter which is introduced into the sediment core from one end, and connected to a ~10 mL syringe from the other end for water collection. Sulfate concentrations were measured using an ion chromatograph 861 Advanced Compact IC from Metrohm with an accuracy of 3%.

### Numerical scheme

To solve numerically the system of 2D advection-diffusion equations, a centered explicit finite difference discretization scheme is used. At time step “n + 1”, the methane 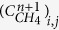 and sulfate 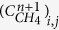 concentrations at nodes (i, j) are calculated from the methane 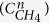 and sulfate 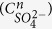 concentrations at time step n and at nodes (i, j), (i − 1, j), (i + 1, j), (i, j − 1) and (i, j + 1) using, for constant molecular diffusion coefficients, the following two discretized equations (4) and (5):


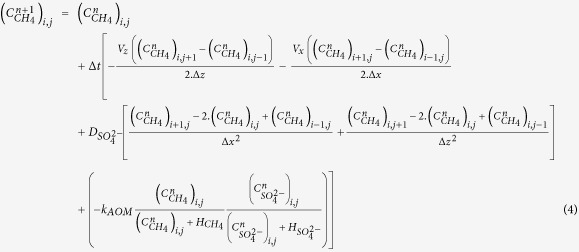



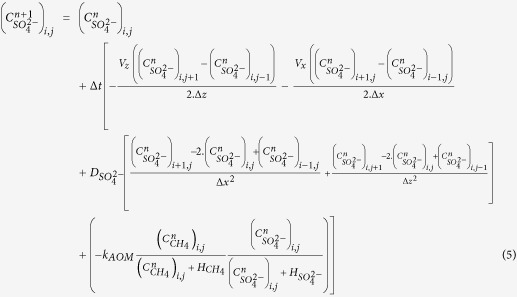


In the previous equations, the subscripts i, and j are space step indices, the superscript n is the time step index and Δ*t*, Δ*x* and Δ*z* are time and space increments, respectively. To carry out the present study, the finite difference numerical scheme was implemented and solved using the Fortran programming language.

## Additional Information

**How to cite this article**: Sultan, N. *et al.* New insights into the transport processes controlling the sulfate-methane-transition-zone near methane vents. *Sci. Rep.* **6**, 26701; doi: 10.1038/srep26701 (2016).

## Supplementary Material

Supplementary Information

## Figures and Tables

**Table 1:** Used nomenclature and parameters of the simulation.

Name	Symbol	Units	Value/reference
Methane molecular diffusion	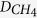	[m^2^/s]	1 10^−9^ 23
Sulfate molecular diffusion	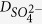	[m^2^/s]	6.3 10^−10^ 23
Methane half–saturation constant	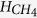	[mM]	1.0 23
Sulfate half–saturation constant	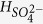	[mM]	0.5 23
Rate constant for AOM	*k*_*AOM*_	[mM/s]	2 10^−9^
