# Supplementary material for: New insights into the transport processes controlling the sulfate-methane-transition-zone near methane vents
Source: Sci Rep. 2016 May 27;6:26701. doi: 10.1038/srep26701 (PMC4882613; doi:10.1038/srep26701)
Supplement: Supplementary Information [file srep26701-s1.pdf]

Supplementary information

New insights into the transport processes controlling the sulfate-methane-transition-zone near methane vents.

By

Nabil Sultan[1*], Sébastien Garziglia[1], Livio Ruffine[1]

*** Corresponding author**

Nabil Sultan

Ifremer, REM/GM/LES, Institut Carnot Ifremer-EDROME

P.O. Box 70, 29280 Plouzané, France

Phone: +33298224259

Email: nabil.sultan@ifremer.fr

Version - April 2016

[1] Ifremer, REM/GM/LES, Institut Carnot Ifremer-EDROME, France.

Supplementary methods

Detection of gas hydrate using Penfeld celerimeter and piezocone.

The detection of gas hydrate shown in Supplementary Figure 1 and Supplementary Figure 2 relied on in situ measurements using the Penfeld penetrometer and analyses of MeBo drill cores. The Penfeld penetrometer allows to acquire continuous profiles of acoustic and geotechnical properties using alternatively a celerimeter or a piezocone mounted at the tip of a up to 30 m long rod. The celerimeter allows to measure compressional wave velocity (Vp) of up to 2000 m/s and attenuation with an input signal frequency of 1 MHz. On in situ acoustic profiles, the presence of hydrate is generally characterized by alternating high and low Vp and increased attenuation which both correlate with marked increases in the load required by the Penfeld penetrometer to push the rod into the ground (Supplementary Figure 1). On in situ geotechnical profiles, the presence of hydrate is marked by simultaneous and high amplitude increases in cone resistance qt, sleeve friction fs, and pore pressure Δu_2[1] (Supplementary Figure 2).

When tying in situ profiles with the seismic profile SY03-THR-PR01, hydrates appear as high amplitude reflections (SP1090 for the acoustic profile and SP 830 for the geotechnical profile- Supplementary Figure 3).

Signature of isolated free gas compartments from seismic data and drilling

Infrared (IR) thermal scanning of the cores immediately after recovery was used as a simple and valuable tool to detect the presence of gas hydrates[2,3]. Indeed, gas hydrate dissociation is an endothermic reaction that cools the sediment. Additionally, the decrease in chlorinity due to the release of fresh water during gas hydrate dissociation was used as a simple and straight-forward method to complement and validate the gas hydrate detection[4]. IR imaging and chlorinity analyses were applied to detect gas hydrates along seismic profile SY03-THR-pr01 where drilling through hydrate and free gas took place[3]. A correlation between seismic data and gas hydrate/free gas occurrences is shown in Supplementary Figure 3. The presence of *in situ* dissolved and free gas as well as gas hydrates led to gas hydrates dissociation and gas bubble growth and exsolution during cores recovery (Supplementary Figure 4). The presence of gaps close to the gas hydrate bearing sediments was observed to fit with *in situ* gas hydrates (dark gray in Supplementary Figure 3) while core intervals with voids to fit with in situ dissolved gas (light gray in Supplementary Figure 3)[3].

During MeBo drillings GMMB11 and GMMB12 gas hydrate chips followed by free gas emissions were clearly observed at depth of 14.7 mbsf and 16.7 mbsf, respectively (Supplementary Figure 3 for GMMB11). The two levels of gas emissions correspond to a diffraction hyperbola's high-amplitude reflectors of Supplementary Figure 3. For GMMB12, sediment-free gas hydrate was encountered below the crossed high amplitude level (brown level in Supplementary Figure 3). In situ piezocone and celerimeter measurements confirmed the presence of sediment-

free gas hydrate below the seismic high amplitude (Figure 1 and Supplementary Figure 3). Based on the above observations, the base of the gas hydrate shown in Figure 3 was considered to fit the seismic high amplitude of SY01-HR-PR01 (Figure 2).

Sensitivity analysis for variation of diffusion coefficients

To determine the impact of the uncertainties in diffusion coefficients for SO_4 and CH_4 through the sediment on the calculation results, an additional run was carried out by taking into account the tortuosity of pores[5]. Supplementary Figure 5 shows the porosity as a function of depth obtained from drill sites GMMB01 and GMMB02 and its effect in terms of tortuosity on the sulfate and methane molecular diffusions. Comparison between model results (lines) and the measured sulfate concentrations (red diamonds) illustrates the role of the tortuosity on the diffusion process and confirms that a lateral advection process of only few decades ago (between 80 and 130 years without tortuosity and between 110 and 175 years with tortuosity) may reproduce the observed results.

References

1. Sultan, N. *et al.* Detection of free gas and gas hydrate based on 3D seismic data and cone penetration testing: An example from the Nigerian Continental Slope. *MAR. GEOL.* **240**, 235-255 (2007).
2. Trehu, A. M. *et al.* Three-dimensional distribution of gas hydrate beneath southern Hydrate Ridge: constraints from ODP Leg 204. *EARTH PLANET. SC. LETT.* **222**, 845-862 (2004).
3. Wei, J. *et al.* Gas hydrate distributions in sediments of pockmarks from the Nigerian margin – Results and interpretation from shallow drilling. *MAR. PETROL. GEOL.* **59**, 359-370 (2015).
4. Ussler, W. & Paull, C. K. Ion exclusion associated with marine gas hydrate deposits. *Natural Gas Hydrates: Occurrence, Distribution, and Detection*, 41-51 (2001).
5. Boudreau, B. P. The diffusive tortuosity of fine-grained unlithified sediments. *GEOCHIM. COSMOCHIM. AC.* **60**, 3139-3142 (1996).

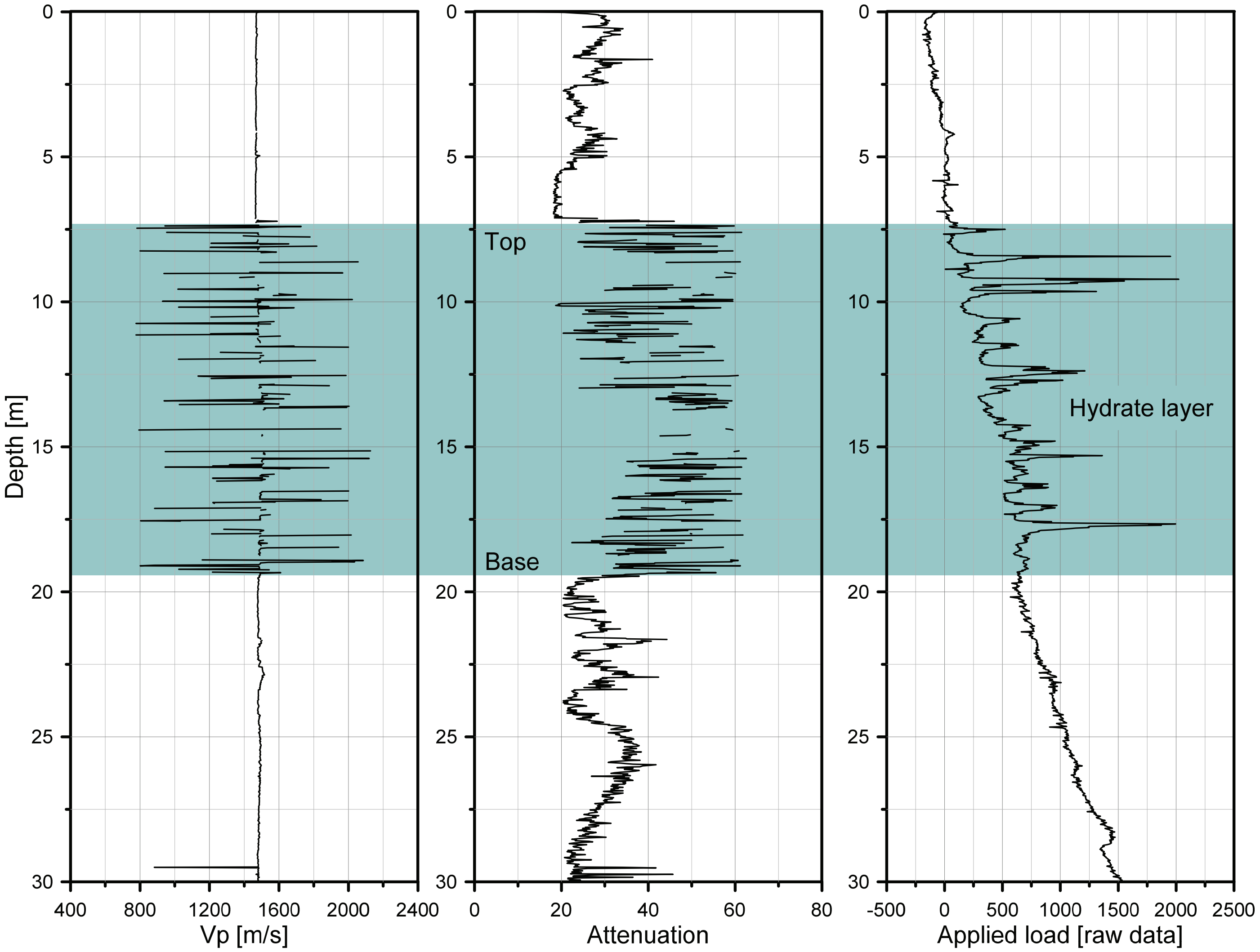

Supplementary Figure 1. Penfeld celerimeter GMPFV03-06 showing the presence of hydrate layer characterized by the alternation of high and low P wave velocities, high attenuation and high applied load to push the Penfeld celerimeter rod. GMPFV03-06 site is at less than 5 m distance from the seismic profile SY03-THR-pr01 (SP 1090). The hydrate layer fit well with the seismic high amplitude shown in Supplementary Figure 3. This figure shows an example of the determination of the top and base of the gas hydrate occurrence zone shown in Figure 1 and Figure 2 based on celerimeter data.

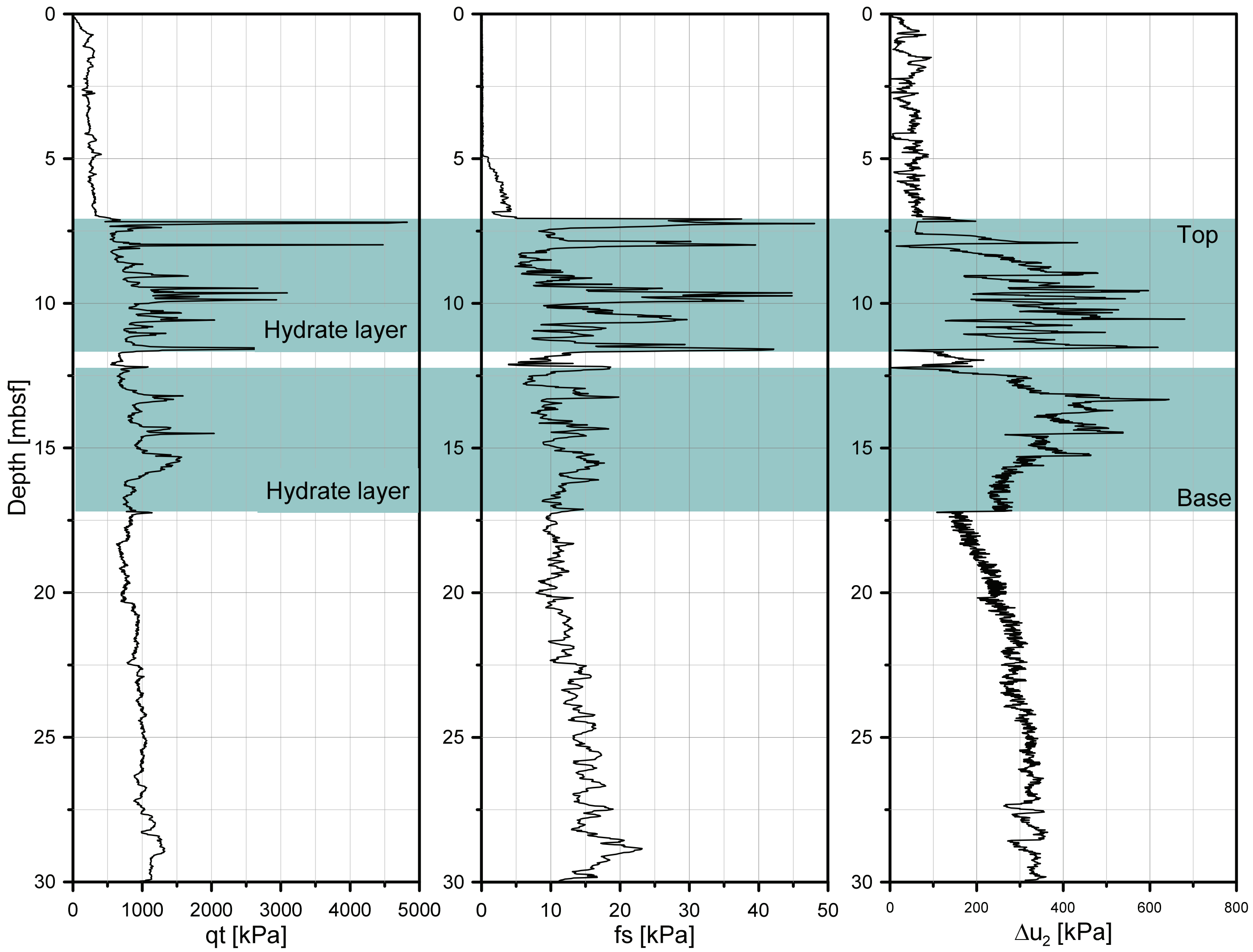

Supplementary Figure 2. Penfeld piezocone CPT02-S08 showing the presence of two hydrate layers characterized by high cone resistance *qt*, friction *fs* and excess pore pressure Δu_2. CPT02-S08 site is at less than 40 m to the SW of seismic profile SY03-THR-pr01 (SP 830). The two hydrate layers fit well with the seismic high amplitude shown in Supplementary Figure 3. This figure shows an example of the determination of the top and base of the gas hydrate occurrence zone shown in Figure 1 and Figure 2 based on piezocone data.

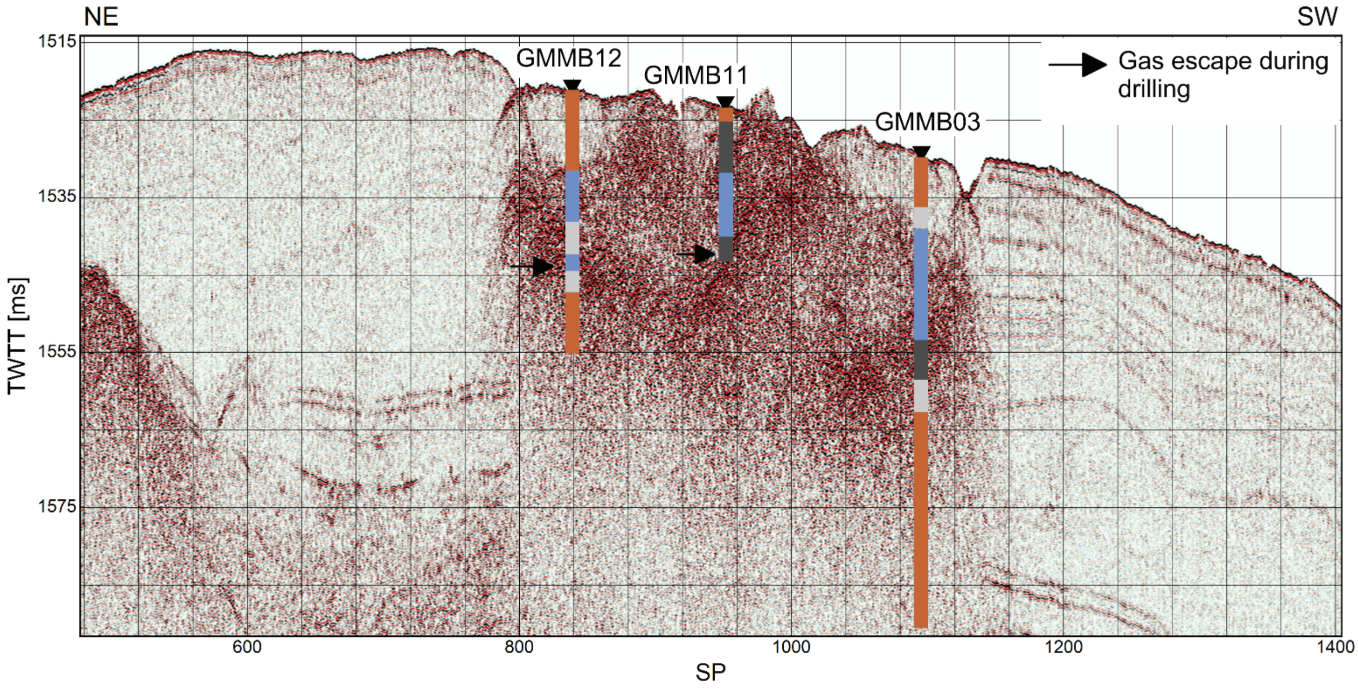

Supplementary Figure 3. Seismic profile SY03-THR-pr01 with MeBo core information of gas hydrates and gas distribution projected. Gas hydrate distribution was interpreted based on the infrared thermal imaging (IR) as well as pore water chloride concentration profile[3]. Colors boxes correspond to: sediment without gas hydrate (brown), sediment with gas hydrate (blue), sediment with high free gas concentration (light grey) and voids (dark grey). Black arrows indicate two levels where gas escape occurred during drilling .

a)

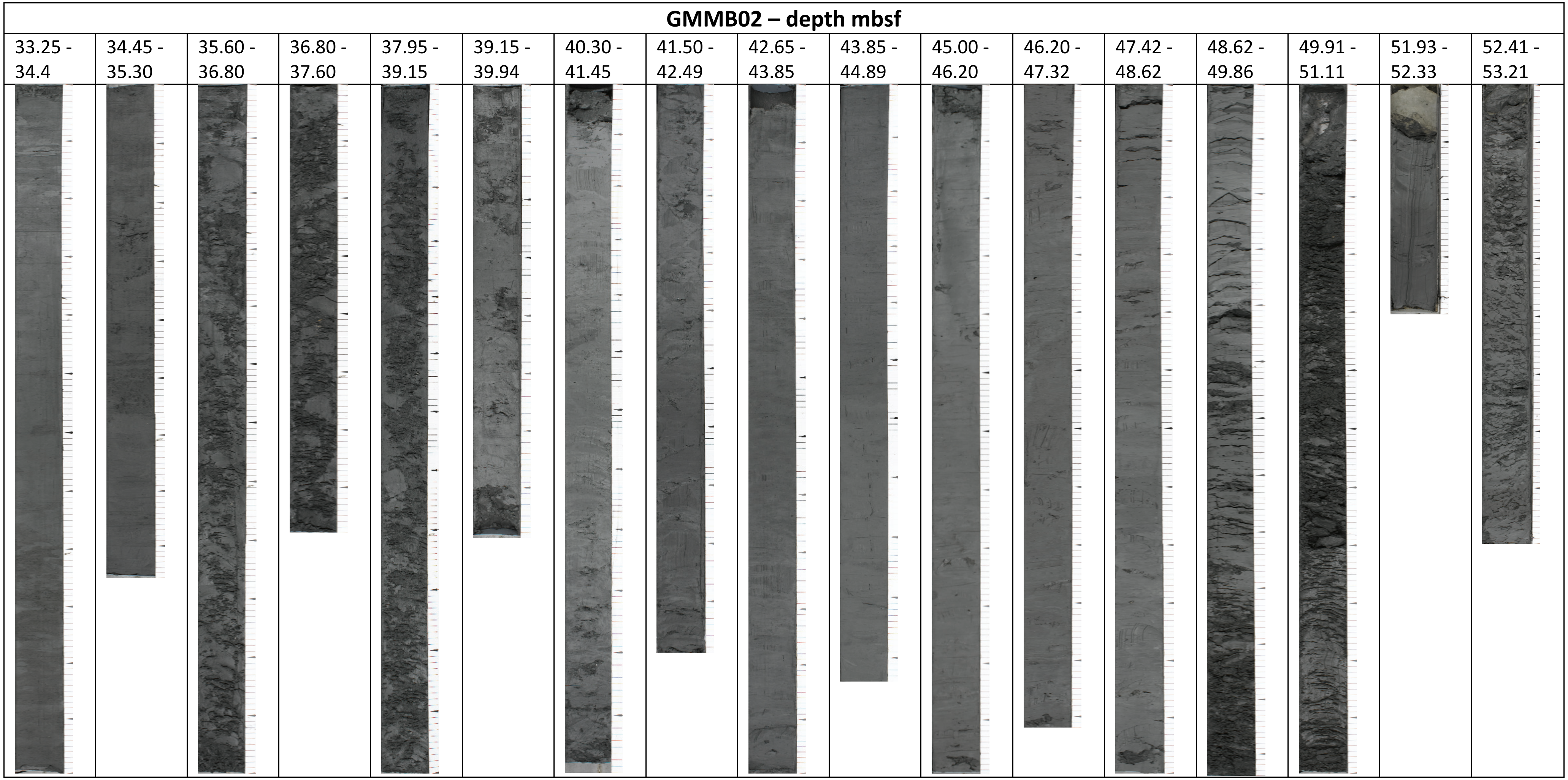
GMMB02 – depth mbsf
33.25 - 34.4
34.45 - 35.30
35.60 - 36.80
36.80 - 37.60
37.95 - 39.15
39.15 - 39.94
40.30 - 41.45
41.50 - 42.49
42.65 - 43.85
43.85 - 44.89
45.00 - 46.20
46.20 - 47.32
47.42 - 48.62
48.62 - 49.86
49.91 - 51.11
51.93 - 52.33
52.41 - 53.21

b)

GMMB06 – depth, mbsf					
0.00 – 1.20	1.20 - 2.45	2.70 - 3.90	3.90 - 4.65	5.05 - 6.25	6.25 - 6.67

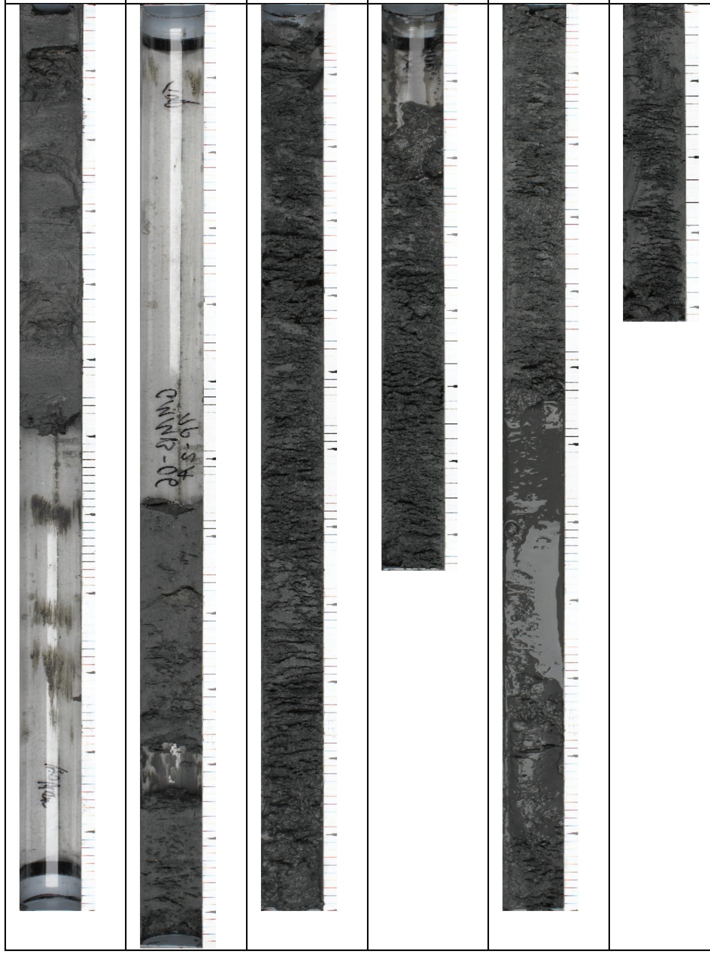

c)

GMMB07 – depth, mbsf							
0.00 – 1.20	1.20 - 1.50	2.70 - 3.90	3.90 - 5.15	5.28 - 6.48	6.48 - 7.62	7.75 - 8.95	8.95 - 10.19

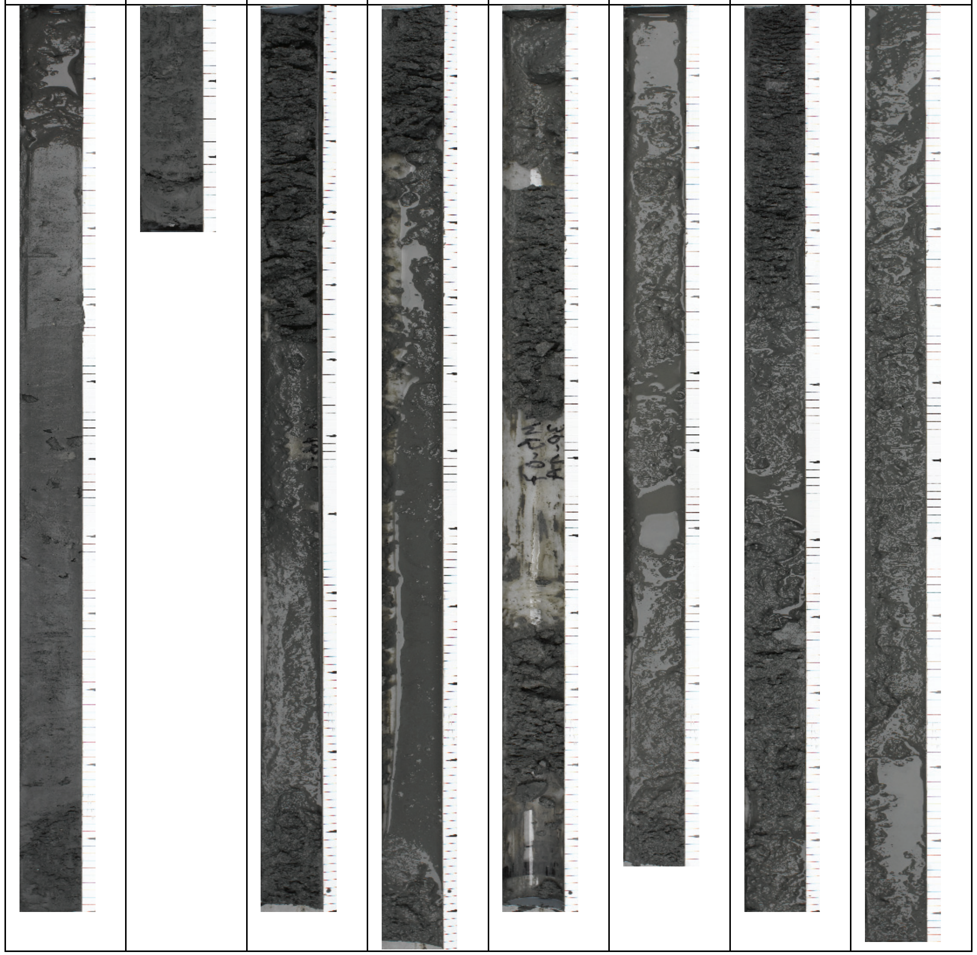

Supplementary Figure 4. Core photos from GMMB02 (a), GMMB06 (b) and GMMB07 (c). Voids in GMMB06 and GMMB07 correspond to sediment expansion/expulsion generated by hydrate dissociation during core recovery.

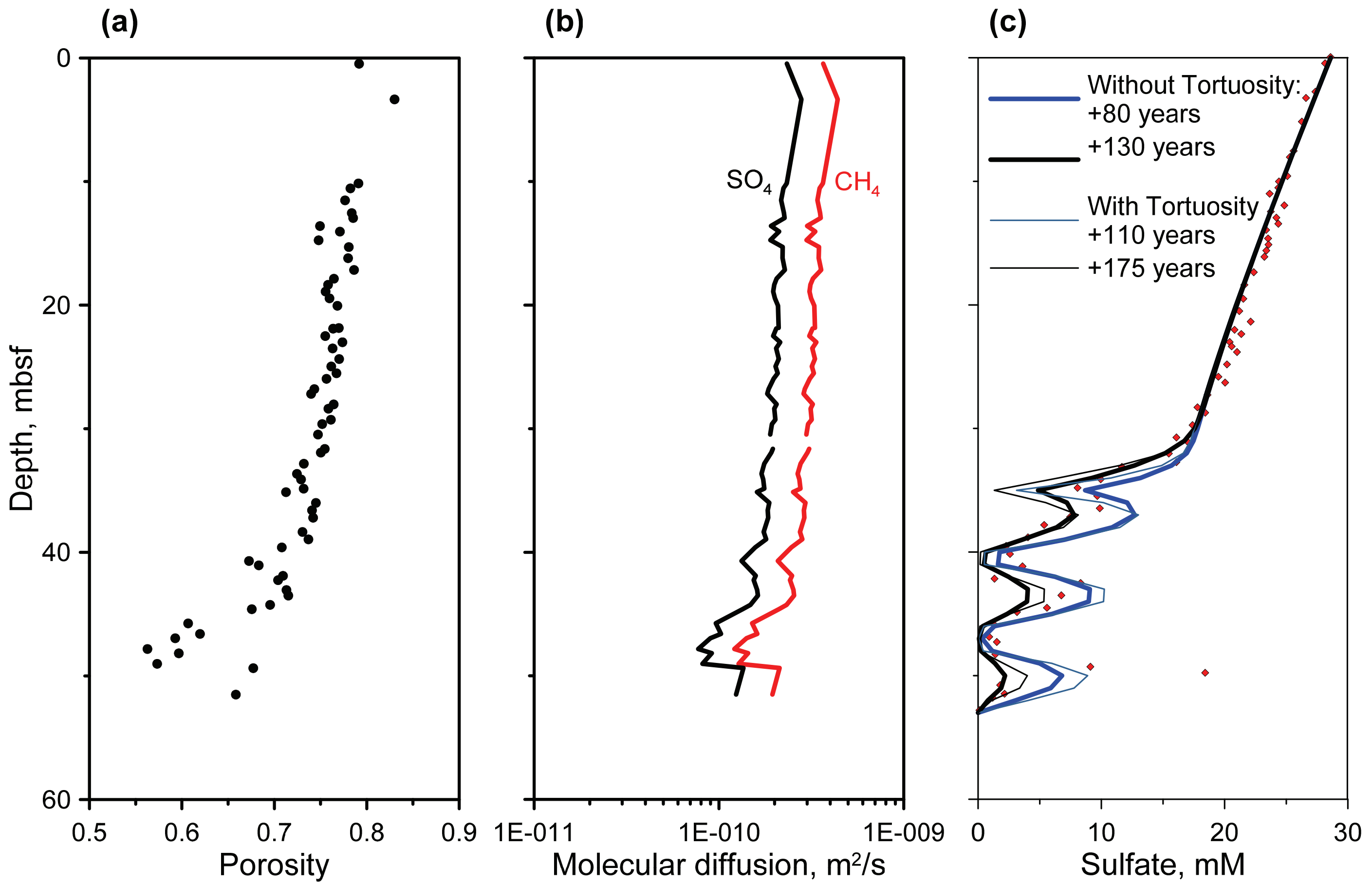

Supplementary Figure 5. Porosity as a function of depth obtained from drill sites GMMB01 and GMMB02 (a) and calculated sulfate and methane molecular diffusions by considering tortuosity[5] (b). Comparison between model results (lines) and the measured sulfate concentrations (red diamonds) illustrates the role of the tortuosity on the diffusion process.
